# Pancreatic pseudocyst with spontaneous cutaneous fistulization: Case report

**DOI:** 10.1097/MD.0000000000012051

**Published:** 2018-08-21

**Authors:** Milan Radojkovic, Predrag Kovacevic, Danijela Radojkovic

**Affiliations:** a Digestive Surgery Clinic; b Internal Medicine Clinic, Clinical Center Nis, Nis, Serbia.

**Keywords:** pancreatic pseudocyst, spotaneous pancreaticocutaneous fistula, conservative treatment

## Abstract

**Rationale:**

Pancreatic fistula occurs as a result of pancreatic duct disruption during acute pancreatitis. An external or pancreatico-cutaneous fistula is defined as a leakage of pancreatic secretion through an abdominal wound or previously inserted drain. If the extravasated pancreatic juice is walled-off by the fibrous or granulation tissue, a pseudocyst is formed. Fistulization of the pancreatic pseudocyst into the different surrounding hollow viscera is reported. However, we present a patient with spontaneous cutaneous fistulization of the pancreatic pseudocyst into the lumbar region successfully treated conservatively. Such an extremely rare presentation is only reported twice and thus highly unexpected during the follow-up of patients after recovery from acute pancreatitis.

**Patient concerns:**

The patient presented with 5-days intermittent fever and a tender, fluctuant, and erythematous swelling of the left lumbar paravertebral region with black necrotic skin spot on the top of it.

**Diagnoses:**

Abdominal computed tomography scan revealed retroperitoneal pseudocyst originating from the pancreatic body and tail and extending to the left flank.

**Interventions:**

Incision of the swelling evacuated dark amylase rich fluid. Colostomy disc and bag were applied to collect further spontaneous outflow of pseudocyst content.

**Outcomes:**

Symptoms instantly resolved and the patient was managed conservatively with ambulatory follow-up of the daily volume of fistula discharge. Over the next 37 days daily fistula output gradually reduced to nil with the spontaneous closure of the external skin fistula opening.

**Lessons:**

Frequent follow-ups of patients after severe acute pancreatitis are necessary for early detection and timely successful treatment of pancreatic pseudocysts with such unusual and rare presentation.

## 1 Introduction

Pancreatic pseudocysts are collections of pancreatic juice leaked out of a disrupted pancreatic duct, localized with non-epithelialized fibrous wall, and usually appear as a complication of acute or chronic pancreatitis. Internal fistulization of pancreatic pseudocysts into the adjacent hollow viscus or cavity such as colon or pleural space is known and well-described.^[1,2]^ External pancreatic fistulas typically occur as a result of pancreatic surgery or percutaneous catheter drainage of pseudocysts. Herein, we present a very rare case of spontaneous cutaneous fistulization of the pancreatic pseudocyst into the lumbar region successfully treated conservatively. Such an extremely unusual pseudocyst presentation must be kept in mind during the follow-up of patients after pancreatitis as it may have important implications for further treatment decision-making. All the procedures, data collection and presentation were approved by the hospital Ethics Committee (Decision No: 452/11) and with the patient's informed consent.

## 2 Case report

**Figure 1:**
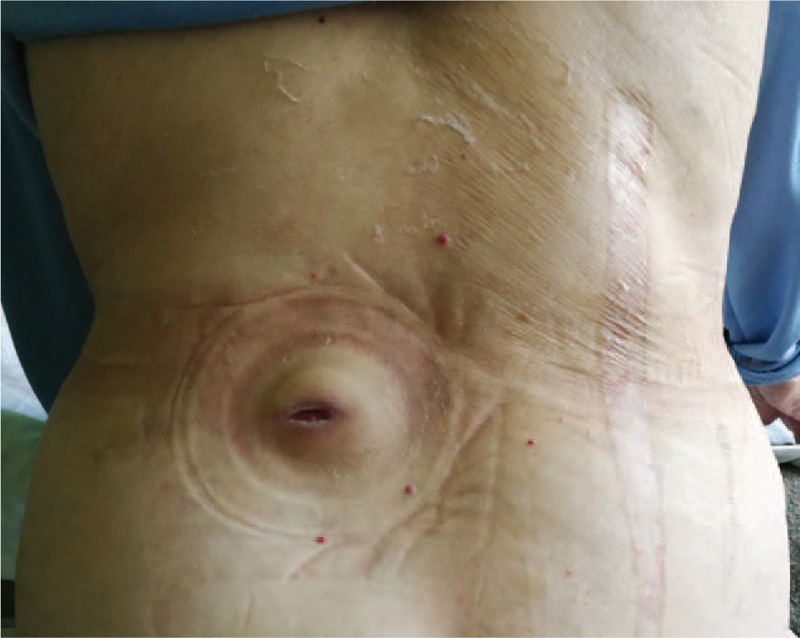
Incised swelling of the left lumbar paravertebral region.

**Figure 2:**
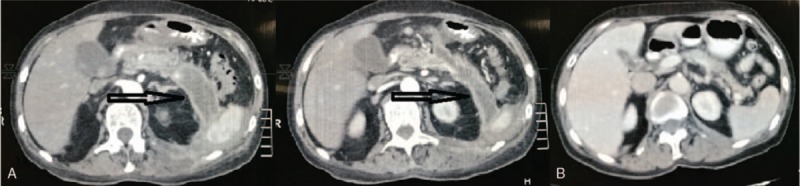
A. Abdominal computed tomography scan depicting retroperitoneal cylinder-shaped fluid collection with thick fibrous wall originating from the pancreatic body and tail and extending to the left flank (arrows). B. Normal follow-up computed tomography scan 2 months after the spontaneous fistula resolution.

A 65-year-old woman presented with 5-day intermittent fever (up to 38 °C) and a 70 × 50 mm tender, fluctuant, and erythematous swelling of the left lumbar paravertebral region with black necrotic skin spot on the top of it (Fig. 1). Previously she was treated at the regional hospital for severe gallstone pancreatitis for 23 days and discharged 2 months ago. She denied any other symptoms over the past 2 months. Abdominal computed tomography scan revealed retroperitoneal cylinder-shaped fluid collection with thick fibrous wall originating from the pancreatic body and tail and extending to the left flank (Fig. 2A). Incision of the swelling through the necrotic skin spot evacuated 350 mL of dark fluid. Amylase level in the fluid was in excess of 24,000 IU. Colostomy disc and bag were applied to collect further spontaneous outflow of pseudocyst content. Fever instantly resolved and the patient was managed conservatively with low-fat diet, oral pancreatic enzyme supplementation, and somatostatin analogue administered and ambulatory follow-up of the daily volume of fistula discharge. Over the next 37 days daily fistula output gradually reduced from initial 140 mL on the first day after the incision to nil with the spontaneous closure of the external skin fistula opening. Patient recovered uneventfully, follow-up computed tomography scan 2 months after the spontaneous fistula resolution was normal (Fig. 2B), and is asymptomatic.

## 3 Discussion

Pancreatic pseudocysts are associated with 10% of patients with acute and 20% to 38% of patients with chronic pancreatitis.^[3]^ They are located intra-abdominally and when they erode adjacent hollow organs or spaces internal pancreatic fistulas occur. An external pancreatic fistula, or pancreatico-cutaneous fistula is, by definition, leakage of the pancreatic juice through the abdominal wound or inserted drain persisting for >7 days and most commonly occurs as a complication of pancreatic surgery or percutaneous drainage of pseudocysts. However, spontaneous external cutaneous fistulization of the pancreatic pseudocyst is extremely rare. To our knowledge only 2 such cases were reported: one with external opening at umbilicus, treated conservatively and second with free communication with the left flank managed at surgery.^[3,4]^

The preferred imaging method for such patients is computed tomography with fistulogram.^[5,6]^ Urgent management of pancreatic pseudocysts is mandatory if complications occur, such as infection, bleeding, rupture or internal fistulization, and mass effect (usually those >6 cm in diameter) and treatment modalities include operative, endoscopic, or percutaneous drainage.^[7]^ Nevertheless, initial treatment is conservative as spontaneous resolution is expected in the majority of cases including those with low-output (<200 mL per day) external fistulas such as our patient.^[8]^ Severe complications such as hydroelectrolyte disturbances, organ failure, or sepsis may occur in patients with high-output external drainage of pancreatic fluid and require more intensive therapeutic measures: nil per os, parenteral nutrition, IV fluids, electrolyte correction, naso-gastric tube placement, analgesics, and antibiotics. Due to good general status and fast resolution of symptoms our patient did not require these measures with exception of daily skin care and protection for mild skin excoriation. Also, gradual reduction and spontaneous closure of the external pancreatic fistula after 37 days was due to the unobstructed downstream flow of the pancreatic juice through the pancreatic duct. Therefore, early endoscopic retrograde pancreatography and stent insertion or surgery including Roux-en-Y pancreatico-jejunostomy or resection were not mandatory to ensure fistula closure.^[9]^ Frequent follow-ups of patients after severe acute pancreatitis are necessary for early detection and subsequent successful treatment of pancreatic pseudocysts with such unusual and rare presentation.

## Author contributions

**Conceptualization:** Milan Radojkovic.

**Data curation:** Milan Radojkovic, Predrag Kovacevic, Danijela Radojkovic.

**Formal analysis:** Milan Radojkovic, Predrag Kovacevic, Danijela Radojkovic.

**Supervision:** Predrag Kovacevic, Danijela Radojkovic.

**Validation:** Predrag Kovacevic, Danijela Radojkovic.

**Writing – original draft:** Milan Radojkovic.

**Writing – review & editing:** Milan Radojkovic.
